# Mapping Cortical Thickness of the Patients with Unilateral End-Stage Open Angle Glaucoma on Planar Cerebral Cortex Maps

**DOI:** 10.1371/journal.pone.0093682

**Published:** 2014-04-07

**Authors:** Piotr Bogorodzki, Ewa Piątkowska-Janko, Jerzy Szaflik, Jacek Paweł Szaflik, Mira Gacek, Paweł Grieb

**Affiliations:** 1 Institute of Radioelectronics, Warsaw University of Technology, Warsaw, Poland; 2 Department of Experimental Pharmacology, Medical Research Centre, Polish Academy of Sciences, Warsaw, Poland; 3 Department of Ophthalmology, Medical University of Warsaw, SPKSO Ophthalmic University Hospital, Warsaw, Poland; 4 Department of Experimental Pharmacology, Medical Research Centre, Polish Academy of Sciences, Warsaw, Poland; University of Montreal, Canada

## Abstract

**Purpose:**

To estimate and compare cerebral cortex thickness in patients with unilateral end-stage glaucoma with that of age-matched individuals with unaffected vision.

**Methods:**

14 patients with unilateral end-stage primary open angle glaucoma (POAG) and 12 age-matched control individuals with no problems with vision were selected for the study based on detailed ophthalmic examination. For each participant 3D high-resolution structural brain T1-weighted magnetization prepared MR images were acquired on a 3.0 T scanner. Brain cortex thickness was estimated using the FreeSurfer image analysis environment. After warping of subjects' cortical surfaces to FreeSurfer common space, differences between POAG and control groups were inferred at the group analysis level with the General Linear Model.

**Results:**

The analysis performed revealed local thinning in the visual cortex areas in the POAG group. Statistically significant differences form 600 mm^2^ clusters located in the Brodmann area BA19 in the left and right hemisphere.

**Conclusion:**

Unilateral vision loss due to end-stage neuropathy from POAG is associated with significant thinning of cortical areas employed in vision.

## Introduction

According to the current definition the term glaucoma describes a group of diseases that involve optic neuropathy determined by characteristic structural change and functional deficit related to the loss of retinal ganglion cells (RGC) which originate in axons that exit the eye and form the optic nerve. The most prevalent type is primary open-angle glaucoma (POAG), distinguished by late onset (typically after the age of 60) and slow progression over months to years. This type of disease is frequently unilateral, and even in the absence of treatment only a minority of patients develop bilateral blindness [1]. Nevertheless, because prevalence of the disease is high (up to 4% of the general population, according to some epidemiological studies [2]), the number of patients blinded by POAG is substantial.

Although in the retina glaucoma selectively affects the layer of RGC, post-mortem histology has revealed that signs of neurodegeneration and associated metabolic changes are not restricted to RGC and the optic nerve, but encompass upstream parts of the central visual pathway including visual cortex. The first report on significantly lower, compared to control, neuronal cell count in the lateral geniculate nucleus [3], was considered controversial at a time [4], but further research confirmed and extended this finding. For example, Gupta et al. [5] reported in the brain of a patient with advanced glaucoma and 50% visual field loss the evidence of degenerative changes in the brain involving the intracranial optic nerve, lateral geniculate nucleus, and visual cortex. More recently, application of various neuroimaging methods (such as T2-weighted imaging, diffusion tensor imaging, functional magnetic resonance and magnetic resonance spectroscopy) to studies of glaucoma patients brains provided further confirmation for involvement of the entire visual pathway in patients suffering of glaucoma, in particular in the advanced stage (see [6]–[10] and reviews [11], [12]).

Recently, voxel-based morphometry (VBM) has been employed to quantitatively describe structural differences between brains of patients with POAG and age-matched subjects with unaffected vision [13]–[18]. VBM is an automated technique of spatial normalization of brain magnetic resonance images of individual subjects on a voxel basis with the use of deformation fields., It is used to quantify the amount of neural tissue in the voxels, permitting voxel-wise parametric statistical assessment of structural brain differences between chosen patient populations [19]. Most of the recent studies confirmed that glaucoma in advanced stage is associated with reduced volume of all structures along the visual pathway, including gray matter of the visual cortex. However, in some of them gray matter reductions were not found in early stage glaucoma [14], whereas in the advanced stage grey matter reductions in visual cortex were accompanied with increased grey matter volume in the neighboring voxels [16]. Moreover, in one recent report [18] all five structures (right and left inferior occipital gyri and the right middle occipital gyrus, right inferior temporal gyrus, and right occipital lobe white matter) which were found significantly different between the glaucoma group compared to the control groups, were larger in the glaucoma group.

Another method that can be used for quantitative assessment and between-group comparisons of cortical structures is based on the surface deformation algorithm. The estimation of cortical thickness is performed with the use of the FreeSurfer software [20]. This approach differs from VBM which uses a volume domain approach as implemented in well-established software platforms, such as SPM or FSL. FreeSurfer uses surface-based analyses of individual subjects accompanied by surface-based registration (SBR). In theory, SBR has an intrinsic advantage over VBM because it respects the topology of the cortical sheet. The accuracy of the thickness measurements derived by this technique has been validated by histological [21] and manual measurements [22].

Importantly, VBM and cortical surface models reveal different aspects of brain cortex anatomy. Because the human brain cortex is gyrencephalic (highly folded and convoluted due to gyri and sulci), grey matter volume is not a linear geometric function of cortical surface area [23]. Moreover, it has been suggested that cortical surface area and cortical thickness, hence also grey matter volume, are independent both globally and regionally [24]. The first two attributes may even be influenced by different, uncorrelated genetic factors [25]. More recently [26], thinning in Brodmann areas BA 17 and BA 19 related to vision was shown in a group of POAG patients. However, that study excluded the end-stage patients.

The aim of our study was to compare cerebral cortex thickness between a group of patients with unilateral end-stage glaucoma and a group of age-matched individuals with unaffected vision using SBR intersubject registration. Additionally, a mapping algorithm showing cortical thickening on flat maps of predefined cortical areas was adopted to improve localization of pathological changes and locate them in cortical atlases similar to ones used in retinotopy studies in functional magnetic resonance imaging (fMRI).

## Materials and Methods

### Ethics statement

The Ethics Committee of the Medical University of Warsaw (MUW) approved the study.

### Subjects

Each subject enrolled in the study provided written informed consent prior to completing an extended set of ophthalmic examination conducted in the Department of Ophthalmology MUW, including visual acuity, refraction, intraocular pressure (IOP) measurement using applanation tonometry, pachymetry, gonioscopy, dilated slit lamp examination and nonmydriatic retinal photography. Visual fields were assessed using standard automated perimetry (HFA 720i -Humphrey Field Analyzer, Carl Zeiss). Scanning laser polarimetry with variable corneal compensation (GDx-VCC; Carl Zeiss Meditec) and confocal scanning laser ophthalmoscopy (Heidelberg Retina Tomograph II [HRT-II]; Heidelberg Engineering, Heidelberg, Germany) were used to measure retinal nerve fiber layer (RNFL) thickness and optic disc topography, respectively.

A group of 14 POAG patients (6 women, 8 men; age 76±7.5) with advanced unilateral changes in glaucomatous eye (no light perception to hand movement, c/d ratio 0.9–1.0) and less affected fellow eye (BCVA in the range 0.5–1.0 and losses in visual field from 30 to 1 dB) was selected. All patients met criteria for POAG according to European Glaucoma Society Guidelines: IOP>21 mmHg at different stages, currently controlled on glaucoma typical medications, an open anterior chamber angle in gonioscopy, glaucomatous optic nerve damage, characteristic visual field loss as damage progresses, absence of signs of secondary glaucoma or a non-glaucomatous cause for the optic neuropathy [27], [28]. The medical records of glaucoma patients were reviewed for date of diagnosis, time of observation, history of topical medications, progression of glaucomatous changes based on visual field and optic disc, history of head and eye trauma, medical history including oral medications review e.g. steroids, previous eye surgeries, family history of glaucoma.

A control group of 12 healthy subjects (HC) matched for age and sex with the POAG group was identified using the following inclusion criteria: BCVA better than 0.5 and perimetry mean deviation (MD) better than −5 dB in both eyes. Basic information on the subjects is shown in Table 1 (all individual data are provided in Supplementary data Table S1).

**Table 1:** Basic Information of Enrolled Subjects.

Patient's group	POAG	HC
Numer of subjects	14	12
Gender	6F/8M	9F/3M
Age	76±7 y	67±10 y
Vison	Unilaterally blind,fellow eye affected less thanMD>−20 dB)	Good vision in both eyesMD>−5 dB

A null hypothesis of equal means of age between POAG (6 female, 8 men; age (mean/SD) 76/7.5) and HC (9 female, 3 men, age (mean/SD) 66.5/9.8) can not be rejected on standard p<0.05 level (T = 1.45, p<0.08), thus age was considered as a confounding factor in subsequent analyses.

All subjects were screened for standard MRI exclusion criteria: no conditions/medications known to affect cerebral metabolism, no metal in the body that could not be removed, and no history of claustrophobia. Visual inspection of MR images by a neuroradiologist revealed no evidence of overt pathology in cortex and subcortical areas.

### Methodology: Brain MRI and Brain Surface Flattening Methodology

MRI images were acquired by a 3-Tesla scanner (Trio; Siemens, Erlangen, Germany) in the Bioimaging Research Center at the Institute of Physiology and Pathology of Hearing in Kajetany, Poland. A High-resolution T_1_-weighted MR volume images were acquired with the Magnetization Prepared RApid Gradient-Echo (MP-RAGE) imaging sequence with the following settings: TR = 2.3 s; TE = 3.0 ms, slice thickness 1.2 mm, 256×240 matrix, FOV = 23.8×25.5 cm, 176 sagittal slices. The original imaging data were fetched from the scanner in the DICOM format and anonymized. The introductory subject-specific analysis comprised of estimation of cortical thickness with the FreeSurfer (FS v5.1.0) image analysis environment (http://surfer.nmr.mgh.harvard.edu/) [29]–[31]. First, the high resolution T1 MPRAGE volumes were converted to FreeSurfer format, normalized for intensity [32] and resampled to isotropic voxels of 1×1×1 mm. Next, the skull was removed using a skull-stripping algorithm and segmented into three tissue types (white matter, grey matter and CSF) [29], [31], [33]–[36].

For each subject the distance between the white-matter and pial surfaces can be sampled at each triangle vertex in the pial mesh [21], [36], [37], colour coded and displayed as a 3D model for presentation purposes. In order to allow group comparisons and draw population specific inferences, an additional second level analysis was performed. Similar to volumetric group analyses [38] a warping of subject cortical surfaces to a reference surface with known localization of cortical structures (electronic atlases) is required. An FS standard average cortical surface template (FSaverage) and nonlinear procedure were used [35] that align cortical folding patterns to the template with a number of deformable procedures including surface inflation and spherical registration minimising cortical geometry mismatch [22], [39], [40]. Finally, subject cortical thickness was resampled at each vertex of the FSaverage pial surface in order to match their resolution and allow subsequent vertex by vertex comparisons. Group-wise differences in cortical thickness between POAG and HC groups were inferred with the aid of a general linear model (GLM) fit of individual thickness maps. Before GLM fitting, normalized cortical thickness measures were smoothed using a full width half maximum (FWHM) Gaussian kernel of 10 mm. The design matrix used in GLM modelling had three regressors: 1) HC group, 2) POAG group, and 3) age as nuisance (no interest) variable.

**Figure 1:**
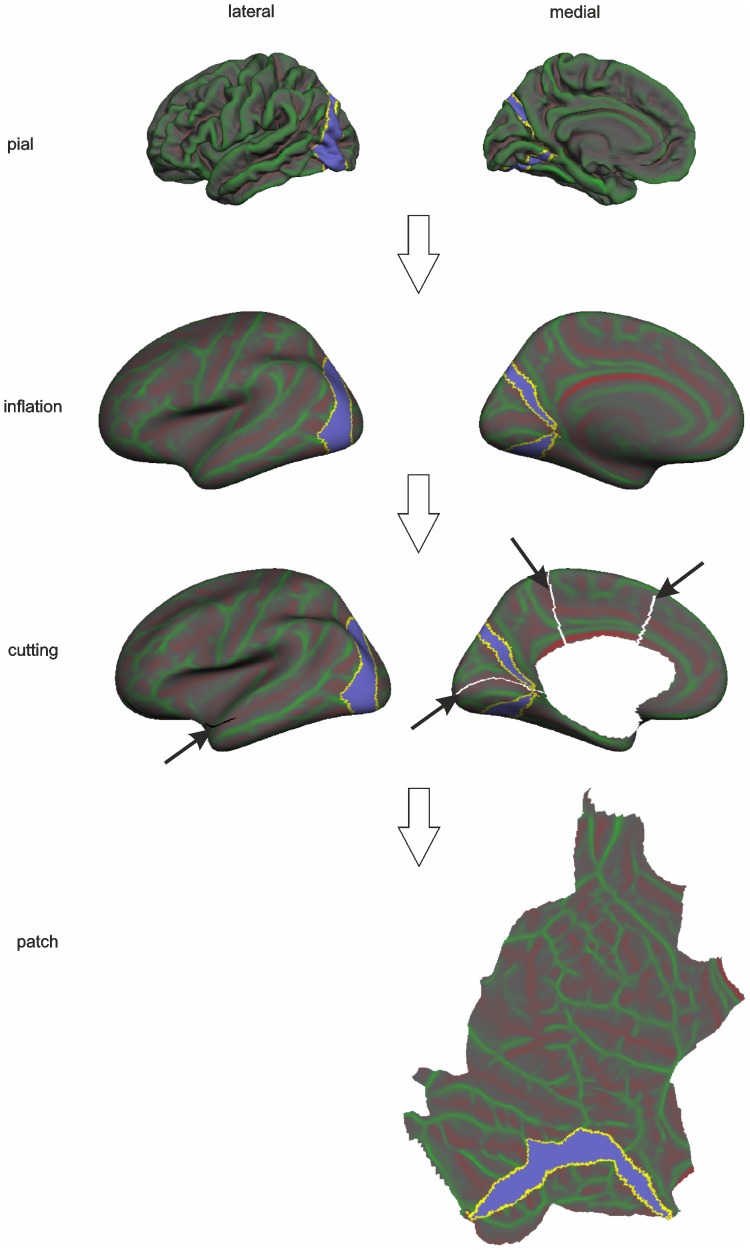
A surface processing pipeline leading to flat maps of the pial surface. A pial surface stored as a triangular mesh was first inflated, then a cortical patch was prepared by removing PCC and making cuts in order to create flat maps of the pial surface. The violet ROI includes Brodmann's 17 area corresponding to primary visual cortex (V1). This is shown for illustration purposes.

Group difference z-maps were corrected for multiple comparisons across vertices using Gaussian-simulation non-parametric inference testing [41]. Results were considered significant at CWP (cluster wise probability) ≤0.001 (10000 simulations, initial cluster-forming threshold at p-uncorrected  = 0.02), fully corrected for multiple comparisons. Clusters of vertices surviving the statistical threshold were projected onto left and right hemispheres of flattened FSaverage pial surfaces. The flattening process of an entire hemisphere is presented in Fig. 1. Additionally, in order to avoid deformation of clusters located on occipital parts of the cortex, a patch covering the visual processing part of the cortex was created. With the aid of flattened cortical maps the localisation of clusters throughout both hemispheres and in relation to those important for visual processing can be readily visualised.

We used three brain atlases to annotate localisation of clusters in both hemispheres. The first one is a cortical parcellation atlas developed by Desikan and Killiany [33], [36] and is called in our paper as “aparc”. It was based on manually labeled cortical regions and consists of 66 cerebral cortex structures (33 structures for each hemisphere) [33]. The second atlas is the Population-Average, Landmark and Surface-based PALS-B12.Visuotopic atlas of human cerebral cortex with 18 vision-related cortical structures (9 structures for each hemisphere) cortex [42], [43]. And the third atlas is the PALS-B12.Brodmann Atlas with 82 cortical structures (41 structures for each hemisphere).

We mapped these structures onto a spherical space to achieve point-to-point correspondence for each subject [36]. The final segmentation of surface-based labelling was based also on a subject-independent probabilistic atlas.

## Results

There was no significant difference in whole brain segmented intracranial volume between POAG (1451.1±213.6 cm^3^) and HC (1464.1±120.3 cm^3^) groups. On cluster-wise whole brain analysis (Table 2) one cluster was detected in the left hemisphere (LH) and two clusters in the right (Table 3). In each case POAG had decreased cortical thickness compared to HC.

**Table 2:** Cluster-wise Cortical Thickness Results in POAG Patients and Control Participants in Left hemisphere.

Cluster No	Max	Size (mm^2^)	TalX	TalY	TalZ	CWP	APARC	PALS-B12 Visuotopic	PALS-B12 Brodmann
1	4.4	778.3	−18.4	−77	−3.3	0.0001	Lingual	VP	BA19

**Table 3:** Cluster-wise Cortical Thickness Results in POAG Patients and Control Participants in Right hemisphere.

Cluster No	Max	Size (mm^2^)	TalX	TalY	TalZ	CWP	APARC	PALS-B12 Visuotopic	PALS-B12 Brodmann
1	5.0	918.0	29.0	−67.3	−4.6	0.0001	Fusiform	V8	BA19
2	3.7	545.9	12.3	−77.1	32.7	0.003	Cuneus		BA19

**Figure 2:**
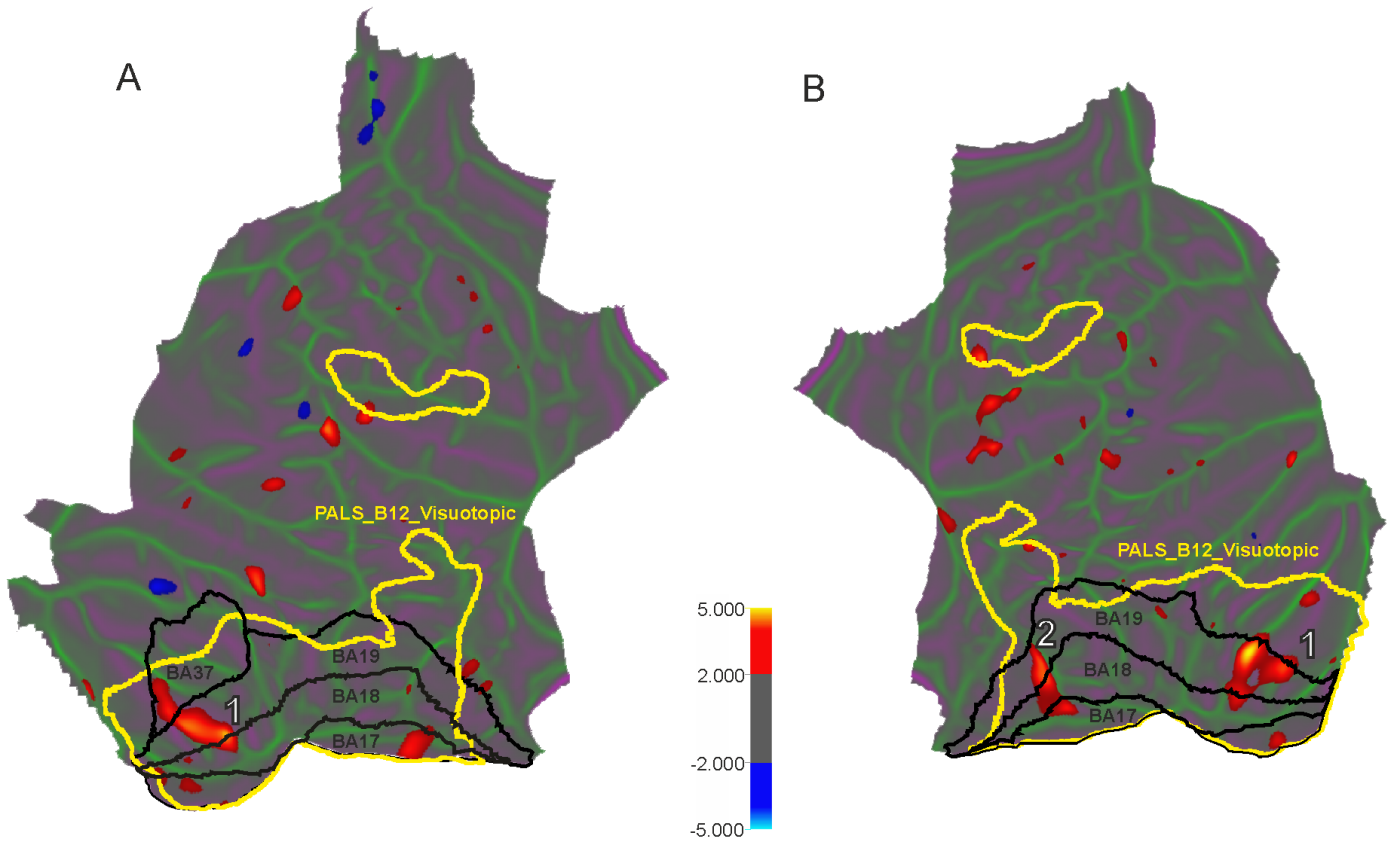
Localization of surface clusters where cortical thickness is significantly reduced in the POAG subjects in comparison to normal controls (p<0.02, uncorrected) for left (A) and right (B) hemispheres. The cold colour represents significantly increased cortical thickness in POAG subjects compared with controls, while the hot colour represents significantly decreased cortical thickness. Yellow lines delineate visuotopic areas taken from PALAIS_B12.Visuotopic cortical atlas. Black lines correspond to the Brodmann areas related to the study. White numbers correspond to clusters surviving the p<0.001 level multiple comparison correction procedure (see Table 2 and 3 for coordinate and cluster size information).

**Figure 3:**
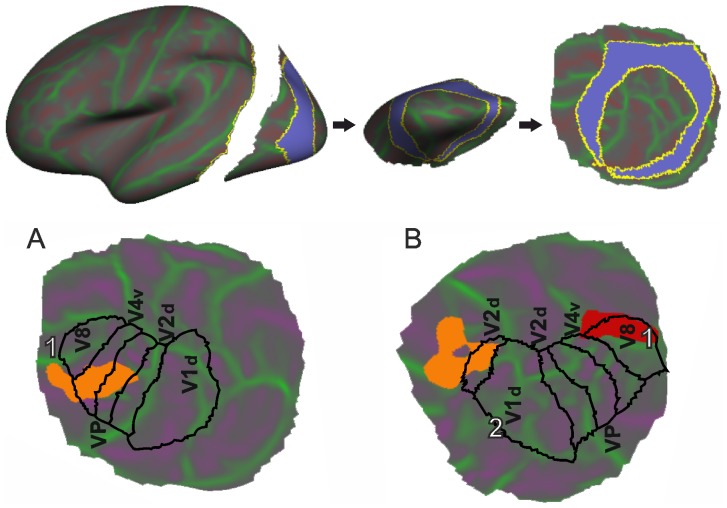
Localization of thinned areas on the visuotopic cortical atlas for left (A) and right (B) hemispheres. Coloured blobs correspond to clusters with statistically significant differences in cortical thickness. A black line corresponds to PALAIS_B12.Visuotopic areas related to the study. White numbers correspond to clusters surviving the p<0.001 level multiple comparison correction procedure (see Table 2 and 3 for coordinate and cluster size information).

In POAG the LH shows decreased cortical (Fig. 2A and Fig. 3A) thickness in 778 mm^2^ cluster localized in BA19 and VP in the lingual gyrus (p<0.0001). There are two clusters in the RH with decreased thickness (Fig. 2B and Fig. 3B): in the fusiform gyrus (918 mm^2^) and cuneus (545.9 mm^2^).

In order to verify our findings, thickness measures were extracted from the atlas based brain regions and statistical analyses were performed to determine which brain regions most differentiated between the two groups. A two sample T-test on group means followed by FDR correction procedure due to multiple comparisons was performed. We confirmed our results using the whole brain analysis for the thickness of left lingual (APARC Atlas), V4v and V1d (PALAIS_B12 Visuotopic Atlas) regions (p<0.01). The thickness of the right precentral gyrus (p<0.01) as well as Vp, and V8 areas were also found to significantly differentiate between the two groups (See Table 4 and 5).

**Table 4:** List of brain ROIs where mean cortical thickness differs significantly between POAG and HC groups (FDR<0.05) in Left hemisphere.

Atlas name	Atlas structure name	FDR corrected p	T
APARC	Lingual	0.005	4.17
PALAIS_B12 Visuotopic	V4v	0.008	3.54
	V1d	0.039	2.37
	V8	0.039	2.37

**Table 5:** List of brain ROIs where mean cortical thickness differs significantly between POAG and HC groups (FDR<0.05) in Right hemisphere.

Atlas name	Atlas structure name	FDR corrected p	T
APARC	Precentral	0.045	3.32
	Lingual	0.047	3.05
	Superiortemporal	0.050	2.51
PALAIS_B12 Visuotopic	Vp	0.006	3.64
	V8	0.012	2.92
	V4v	0.012	2.89
	V1d	0.027	2.41

## Discussion

The main finding of the present study is that patients with unilateral end-stage open angle glaucoma compared to subjects with apparently unaffected vision display statistically significant thinning of some regions of the visual cerebral cortex. These clusters are located in the Brodmann area BA19 in the left hemisphere and in BA19 in the right hemisphere and include areas that are functionally related to vision.

Using the SBR approach, Salat and collaborators [39] have found that in non-demented individuals global thinning of cerebral cortex occurs, evident already by middle age, and that some cortical areas are more affected than others. In that pivotal study one of the particularly affected areas was calcarine cortex near primary visual cortex. Regional heterogeneity in the general volume loss in the human brain during healthy aging has been recently confirmed [44]. In our study, there was only a slight, not statistically significant difference in the age of glaucoma patients and control individuals, therefore we consider it highly unlikely that the thinning of primary visual cortical areas are related to the age difference between the groups and not to the unilateral blindness in the POAG group.

Our data are consistent with the recently reported analysis of cortical thickness in POAG patients compared to age matched controls [26]. In that report the groups compared were comprised of patients and controls with an average age of 46.5 years, and the image processing approach used was similar but not identical to that employed in our study. The patients showed significant bilateral cortical thinning in the anterior half of the visual cortex around the calcarine sulci (left BA 17 and BA 18, right BA17) and in some smaller regions located in the left middle temporal gyrus (BA37) and fusiform gyrus (BA19), but no cortical thickening was found. Differences between our data and those reported by Yu and colleagues [26] may reflect differences in characteristics and in the heterogeneity of patient groups, but may also be a consequence of the modest accuracy of image processing and averaging techniques employed.

Furthermore, our results qualitatively corroborate reports in which the VBM method has been used and reduced visual cortex volume has been found in advanced glaucoma patients [15]. In one VBM study [16] patients with advanced glaucoma displayed not only grey matter reductions in visual cortex, but grey matter volume increases in the neighboring voxels as well. Although patients in that study suffered from advanced glaucoma, they were aged 40 to 50 years, therefore distinctly younger than all but two of our glaucoma patients. Some degree of cross-modal plasticity after late loss of sight has been found in patients of ages between 22 to 60 years [45], and it is tempting to assume that brains of the age 40 to 50 years could balance visual deprivation by crossmodal reorganization leading to compensatory expansion of some cortical areas, eg. auditory cortex, whereas older brains (age >70 years) may not be able to compensate.

Signs of visual cortex degeneration observed in advanced glaucoma have been considered a result of cortical plasticity in response to deprivation of visual stimuli [17], or a consequence of anterograde trans-synaptic diffusion of hypothetical death signals triggered by RGC degeneration [12]. Another intriguing possibility is that in advanced glaucoma cortical degeneration may be a primary neurodegenerative process, followed by trans-synaptic retrograde diffusion of death signals from the visual cortex downstream. The possibility of retrograde neurodegeneration in the visual pathway has been confirmed clinically by Jindahra and colleagues [46], who found that acquired unilateral damage to the occipital lobe resulting in homonymous hemianopia produced RGC loss detectable by optical coherence tomography; interestingly, congenital hemianopia did not result in similar RGC loss. Trans-synaptic degeneration was also considered a possible contributor to retinal cell degeneration in multiple sclerosis by Green and colleagues [47].

Although in glaucoma RGC loss is closely correlated with loss of vision (see, e.g., [48]), there is some indirect evidence that functional deficiency of the visual pathway may precede its structural degradation. Whereas retinal ganglion cell loss is irreversible, functional deficiency in early glaucoma may be reversed, for example by treatment with the phospholipid precursor citicholine [49], [50]. It remains to be investigated whether visual cortex thinning is an early or late event in POAG, and whether it can be reversed, at least partially, by pharmacological or other treatments.

## Supporting Information

Table S1 **A detailed information about subjects enrolled in the study.**
(DOCX)
