# Supplementary material for: Mapping Cortical Thickness of the Patients with Unilateral End-Stage Open Angle Glaucoma on Planar Cerebral Cortex Maps
Source: PLoS One. 2014 Apr 7;9(4):e93682. doi: 10.1371/journal.pone.0093682 (PMC3977872; doi:10.1371/journal.pone.0093682)
Supplement: Table S1 — A detailed information about subjects enrolled in the study. (DOCX) [file pone.0093682.s001.docx]

|  |  | Data for Right Eye (OD) | | | | Data for Left Eye (OS) | | | |
| --- | --- | --- | --- | --- | --- | --- | --- | --- | --- |
| POAG | | BCVA  For distance | BCVA  For near | IOP | MD (dB) | BCVA  For distance | BCVA  For near | IOP | MD (dB) |
| 1 | M9 | 0 | 0 | 12 | (-) | 0.4 | 0.5 | 10 | -29.2 |
| 2 | M3 | 0 | 0 | 4 | (-) | 0.7 | 0.5 | 13 | -29.63 |
| 3 | M10 | 0 | 0 | 52 | (-) | 0.5 | 0.5 | 10 | -8.56 |
| 4 | M8 | 0 | 0 | 10 | (-) | 0.8 | 0.5 | 12 | -5.97 |
| 5 | M11 | ~~*~~  Perception of light | 0 | 14 | (-) | 0.5 | 0.5 | 12 | -14.67 |
| 6 | M5 | ~~*~~  Hand movement | 0 | 12 | (-) | 1.0 | 0.5 | 14 | -6.68 |
| 7 | M4 | ~~*~~  Hand movemen | 0 | 10 | (-) | 0.7 | 0.5 | 9 | -0.72 |
| 8 | M1 | 1.0 | 0.5 | 12 | -3.04 | ~~*~~  Hand movement | 0 | 15 | (-) |
| 9 | M12 | 1.0 | 0.5 | 14 | -1.12 | ~~*~~  Hand movement | 0 | 12 | (-) |
| 10 | M7 | 0.6 | 0.5 | 14 | -9.08 | ~~*~~  Hand movement | 0 | 21 | (-) |
| 11 | M6 | 0.7 | 0.5 | 10 | -17.18 | 0 | 0 | 2 | (-) |
| 12 | M2 | 0.7 | 0.5 | 12 | -3.39 | 0 | 0 | 10 | (-) |
| 13 | M26 | 1.0 | 0.5 | 18 | -1.3 | 1.0 | 0.5 | 18 | -1.1 |
| 14 | M27 |  |  |  |  |  |  |  |  |
| CONTROL | | BCVA  For distance | BCVA  For near | IOP | MD (dB) | BCVA  For distance | BCVA  For near | IOP | MD (dB) |
| 1 | M20 | 0.7 | 0.5 | 15 | - 3,13 | 0.7 | 0.5 | 14 | - 1,19 |
| 2 | M22 | 0.8 | 0.5 | 10 | - 1,84 | 0.05 | 3,6 | 11 | (-) |
| 3 | M25 | 1.0 | 0.5 | 12 | 0.36 | 1.0 | 0.5 | 12 | 0.73 |
| 4 | M23 | 0.8 | 0.5 | 15 | -1.14 | 0.8 | 0.5 | 14 | -0.47 |
| 5 | M19 | 0.5 | 0.5 | 10 | - 2,09 | 0.5 | 0.5 | 10 | - 1,21 |
| 6 | M17 | 0.8 | 0.5 | 13 | -4.36 | 0.7 | 0.5 | 15 | -5.72 |
| 7 | M28 |  |  |  |  |  |  |  |  |
| 8 | M24 | 0.8 | 0.5 | 10 | -3.61 | 0.7 | 0.5 | 10 | -4.03 |
| 9 | M15 | 1.0 | 0.5 | 13 | -0.86 | 1.0 | 0.5 | 14 | -0.19 |
| 10 | M14 | 1.0 | 0.5 | 14 | -0.1 | 1.0 | 0.5 | 13 | -0.24 |
| 11 | M16 | 1.0 | 0.5 | 18 | -1.99 | 1.0 | 0.5 | 18 | -2.91 |
| 12 | M21 | 0.8 | 0.5 | 12 | -1.41 | 0.6 | 0.5 | 13 | -1.46 |

Table S1. A detailed information about subjects enrolled in the study
